# Chronic Periodontal Disease increases risk for Prostate Cancer in Elderly individuals in South Korea: a Retrospective Nationwide Population-based Cohort Study

**DOI:** 10.7150/jca.45369

**Published:** 2020-05-25

**Authors:** Do-hyung Kim, Seong-Nyum Jeong, Jae-Hong Lee

**Affiliations:** Department of Periodontology, Daejeon Dental Hospital, Institute of Wonkwang Dental Research, Wonkwang University College of Dentistry, Daejeon, Korea.

**Keywords:** Cohort studies, periodontal disease, periodontitis, prostate cancer, risk factors

## Abstract

**Objectives:** The association between prostate cancer (PC) and chronic periodontal disease (PD) has been evaluated in previous studies, but results have been inconsistent. This study aimed to determine whether the presence of chronic PD in old age increases the risk of PC using data in the large-scale elderly cohort.

**Materials and Methods:** This nationwide population-based cohort study examined data of 121,240 South Korean individuals aged ≥ 60 years from the National Health Insurance Service-Elderly Cohort database who completed a national program between 2002 and 2015. For a maximum 10 years' observation period, patients with incident PC with chronic PD compared with those without chronic PD were retrospectively tracked, and Cox proportional hazard ratios and 95% confidence intervals (CIs) were calculated, adjusted for potential confounding factors, including age, household income, insurance status, Charlson Comorbidity Index, hypertension, diabetes mellitus, cerebral infarction, angina pectoris, myocardial infarction, prostatitis, smoking status, daily smoking, alcohol intake habits, one-time alcohol intake, and regular exercise.

**Results:** The overall incidence of PC with chronic PD in 10 years was 3.0% (*n* = 2,063). In the multivariate Cox analysis with adjustment for confounding factors, chronic PD was associated with a 24% higher risk of PC (95% CI = 1.16-1.32, *P* < 0.001).

**Conclusion:** Our results suggest that chronic PD is significantly and positively associated with PC. Larger and better-controlled studies are needed to strengthen this evidence of association and explain the underlying biological mechanisms.

## Introduction

Prostate cancer (PC) is one of the most common age-related malignancies in men worldwide and accounts for 20% of new male cancers in the United States in 2019 1. The rate of deaths from PC is decreasing annually but accounts for 9.8% of deaths from male cancer since 2018 and PC is still a common and significant cause of morbidity and mortality in elderly men 1,2. In contrast, in 2016, PC was the fourth most common cancer in South Korea after stomach, lung, and colon cancers, with a total of 11,800 cases, accounting for 9.8% of all male cancers 3. Especially, recently in South Korea, PC has increased incidence by a greater margin than any other male cancer 3.

Although PC is an extremely popular cancer in elderly men, various factors are intricately intertwined with each other, so the primary cause is still controversial 4,5. Congenital factors, such as genetic abnormalities, are closely related to PC, but acquired environments, such as old age and obesity, are also considered important risk factors 6. Additionally, chronic inflammation is also known to play a decisive role in the initiation, promotion, malignant conversion, invasion, and metastasis of cancer, and the evidence that chronic inflammation is the cause of PC has been reported by epidemiological or pathological studies 7-10.

Chronic periodontal disease (PD) is a representative and typical bacterial and inflammatory disease developing within the oral cavity that results in alveolar bone loss and subsequent loss of teeth for a relatively long period 11,12. Several risk factors might be responsible for chronic PD, especially smoking, obesity, and diabetes mellitus, which are also major risk factors or risk indicators for chronic PC 13.

Recently, several studies have carefully reported that PC and chronic PD are closely related and correlated with underlying chronic inflammatory processes 14-16. It is clear that the risk of PC and chronic PD increases with age, and it is considered that patients with chronic PD are more likely to develop PC, but studies are limited. Therefore, this study aimed to determine whether the presence of chronic PD in old age increases the risk of PC using the large-scale elderly cohort database in South Korea.

## Materials and Methods

### Source of data

The study analyses used the National Health Insurance Service-Elderly Cohort (NHIS-EC) database in South Korea, which was established by the NHIS (NHIS sharing service, https://nhiss.nhis.or.kr) to assess the risk and prognostic factors of geriatric disease. In this database, 10% of elderly participants, who were selected from approximately 5.5 million of elderly individuals aged ≥ 60 years who are eligible for national health insurance and medical benefits, are included. Data from 2002 to 2015 (14 years), including sociodemographic information, records of medical history and health examination, and information on nursing institutions and long-term care services, were entered in a retrospective cohort form that does not contain personally identifiable or sensitive medical information.

This study conformed to the Strengthening the Reporting of Observational Studies in Epidemiology (STROBE) guidelines for reporting observational cohort studies (www.strobe-statement.org) and was approved by the Institutional Review Board of Daejeon Dental Hospital, Wonkwang University (approval no. W2003/001-001). The requirement for written informed consent was waived by the NHIS (REQ0000034029).

### Study population

**Figure 1:**
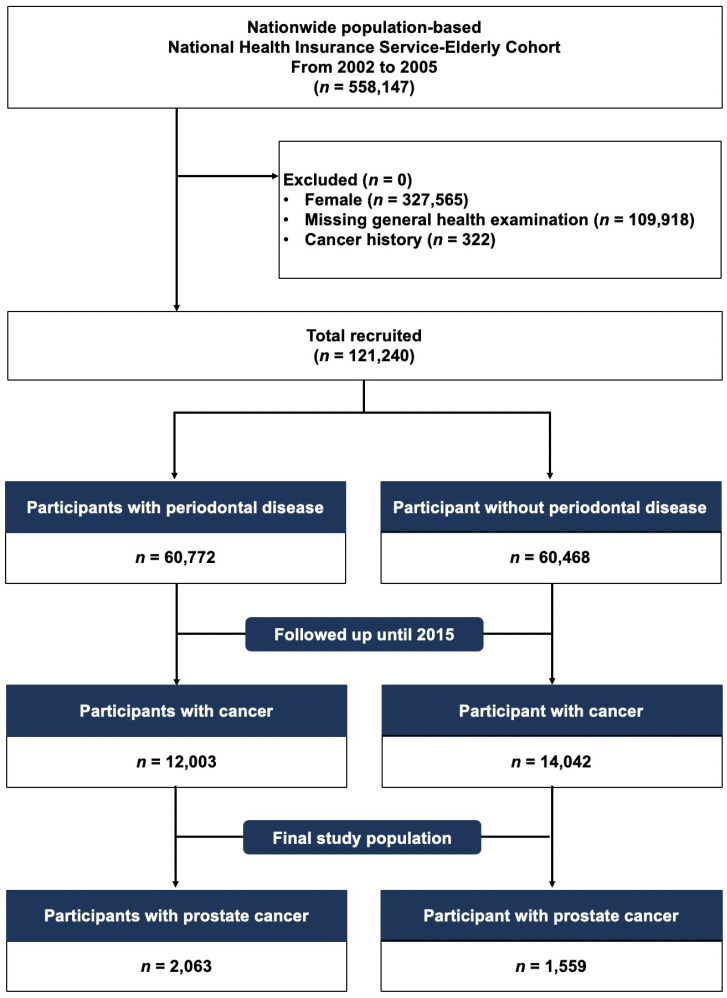
CONSORT flowchart showing the study cohort enrollment process.

We selected the elderly population from those who were included in the NHIS-EC database between January 2002 and December 2005 (*n* = 558,147). We identified eligible participants (*n* = 121,240) after excluding (1) female (*n* = 327,565), (2) those who had missing general and oral health examinations (*n* = 109,918), and (3) those who had a previous history of cancer or new cancer diagnosis or death between 2002 and 2005 (*n* = 322). Then, included participants were classified into a group with chronic PD (*n* = 60,772) and group without chronic PD (*n* = 60,468) and followed up for PC diagnosed until December 31, 2015 (Figure 1).

### Definitions

The chronic PD diagnosis was based on the K052-K056 codes of the Korean Classification of Disease 7th revision (KCD-7), corresponding to codes of the International Classification of Disease 10th revision (ICD-10), according to criteria of the Centers for Disease Control and Prevention/American Academy of Periodontology 17,18. During the oral health examination, chronic PD was assessed and scored based on measurements of clinical periodontal parameters, including bleeding on probing, probing depth, clinical attachment level, and periodontal tooth loss 19. Moreover, to confirm chronic PD, the participants were limited to those diagnosed with chronic PD at least twice during the oral health examination between 2002 and 2005. The primary endpoint was the newly diagnosed PC in 10 years from 2006 to 2015, which was defined using the KCD-7 code C61, corresponding to ICD-10 code C61, by medical oncologists or urologists.

### Covariates

Sociodemographic information, general and oral health examinations, and self-reported questionnaires were used to collect data at the time of enrollment for the following potential variables regarded as risk factors or indicators for PC and included as covariates in the univariable and multivariable analyses: age (two groups: those aged 60-69 years and those aged ≥ 70 years), household income (five groups: those divided into five quintiles based on the insurance fee imposed on each household, with the Medical Aid Program [MAP] group classified into the first quintile), insurance status (three groups: those in the MAP and those in the NHIS groups [self-employed and employees]), Charlson Comorbidity Index (CCI, three groups: those divided into three scores and a brief description of the terms related to CCI is provided in Supplementary Material 1), hypertension (KCD-7 codes I10 and I15, corresponding to ICD-10 codes I10 and I15), diabetes mellitus (KCD-7 codes E10-E14, corresponding to ICD-10 codes E10-E14), cerebral infarction (KCD-7 codes I63-I66, corresponding to ICD-10 codes I63-I66), angina pectoris (KCD-7 codes I20, corresponding to ICD-10 codes I20), myocardial infarction (KCD-7 codes I21 and I22, corresponding to ICD-10 codes I21 and I22), prostatitis (KCD-7 code N41, corresponding to ICD-10 code N41), smoking status and number of cigarettes per day, alcohol intake habit and amount of alcohol intake at a time, and regular exercise per week.

### Statistical analysis

Categorical data were shown as frequencies and percentages. Cohort-related variables, including sociodemographic factors (age, household income, and insurance status), comorbidities (CCI, hypertension, diabetes mellitus, cerebral infarction, angina pectoris, myocardial infarction, and prostatitis), self-reported questionnaire (smoking status, daily smoking, alcohol intake habits, one-time alcohol intake, and regular exercise), were analyzed using the Pearson chi-square test. Hazard ratios (HRs) with 95% confidence intervals (CIs) were calculated via statistical analysis using Cox proportional-hazards regression models after adjusting for sociodemographic factors, comorbidities, and self-reported questionnaire outcomes. The cumulative incidence of PC with and without chronic PD was estimated using the Kaplan-Meier method and compared using the log-rank test. All statistical analyses were conducted using the Statistical Analysis System software version 9.4 (SAS Institute, Cary, NC, USA) and R version 3.5.3 (The R Foundation for Statistical Computing, Vienna, Austria). Statistical significance was considered at two-sided *P*-value <0.05.

## Results

### Baseline characteristics of the study population

Table 1 shows the baseline characteristics of the study population according to the presence and absence of chronic PD. Participants with chronic PD were more likely to have comorbidities, such as hypertension (66.9%), diabetes mellitus (37.5%), angina pectoris (4.2%), myocardial infarction (20.3%), and prostatitis (11.8%), and less likely to smoke (29.5%) and perform regular physical activity (53.4%) compared with those without chronic PD.

### Cumulative incidence of PC

Table 2 shows the cumulative incidence of PC during the follow-up period after diagnosis of chronic PD. During the study period, of participants with chronic PD (*n* = 2,063), 1,487 (72.1%) had hypertension, 827 (40.1%) had diabetes mellitus, 490 (23.8%) had cerebral infarction, 83 (4.0%) had angina pectoris, 560 (27.1%) had myocardial infarction, and 465 (22.5%) had prostatitis. In contrast, in those without chronic PD (*n* = 1,559), 1,033 (66.3%) had hypertension, 553 (35.5%) had diabetes mellitus, 331 (21.2%) had cerebral infarction, 66 (4.2%) had angina pectoris, 303 (19.4%) had myocardial infarction, and 307 (19.7%) had prostatitis.

### Risk of PC

Table 3 shows the univariable and multivariable analyses, with adjustment for sociodemographic factors, comorbidities, smoking, alcohol consumption, and physical activity. The results of the multivariable analysis indicated that the incidence of PC was significantly positively correlated with older age (≥ 70 years, adjusted HR 1.37, 95% CI 1.27-1.48, *P* < 0.001), higher household incomes (fourth and fifth quintiles, adjusted HR 1.21, 95% CI 1.08-1.35, *P* < 0.001 and adjusted HR 1.28, 95% CI 1.15-1.42, *P* < 0.001, respectively), higher CCI score (> 2, adjusted HR 1.19, 95% CI 1.10-1.29, *P* < 0.001), hypertension (adjusted HR 1.13, 95% CI 1.05-1.22, *P* = 0.001), diabetes mellitus (adjusted HR 1.08, 95% CI 1.00-1.16, *P* = 0.041), angina pectoris (adjusted HR 1.24, 95% CI 1.14-1.34, *P* < 0.001), prostatitis (adjusted HR 1.92, 95% CI 1.77-2.08, *P* < 0.001), and current smoking (adjusted HR 1.24, 95% CI 1.14-1.33, *P* < 0.001).

### Association of PD with incidence of PC

**Figure 2:**
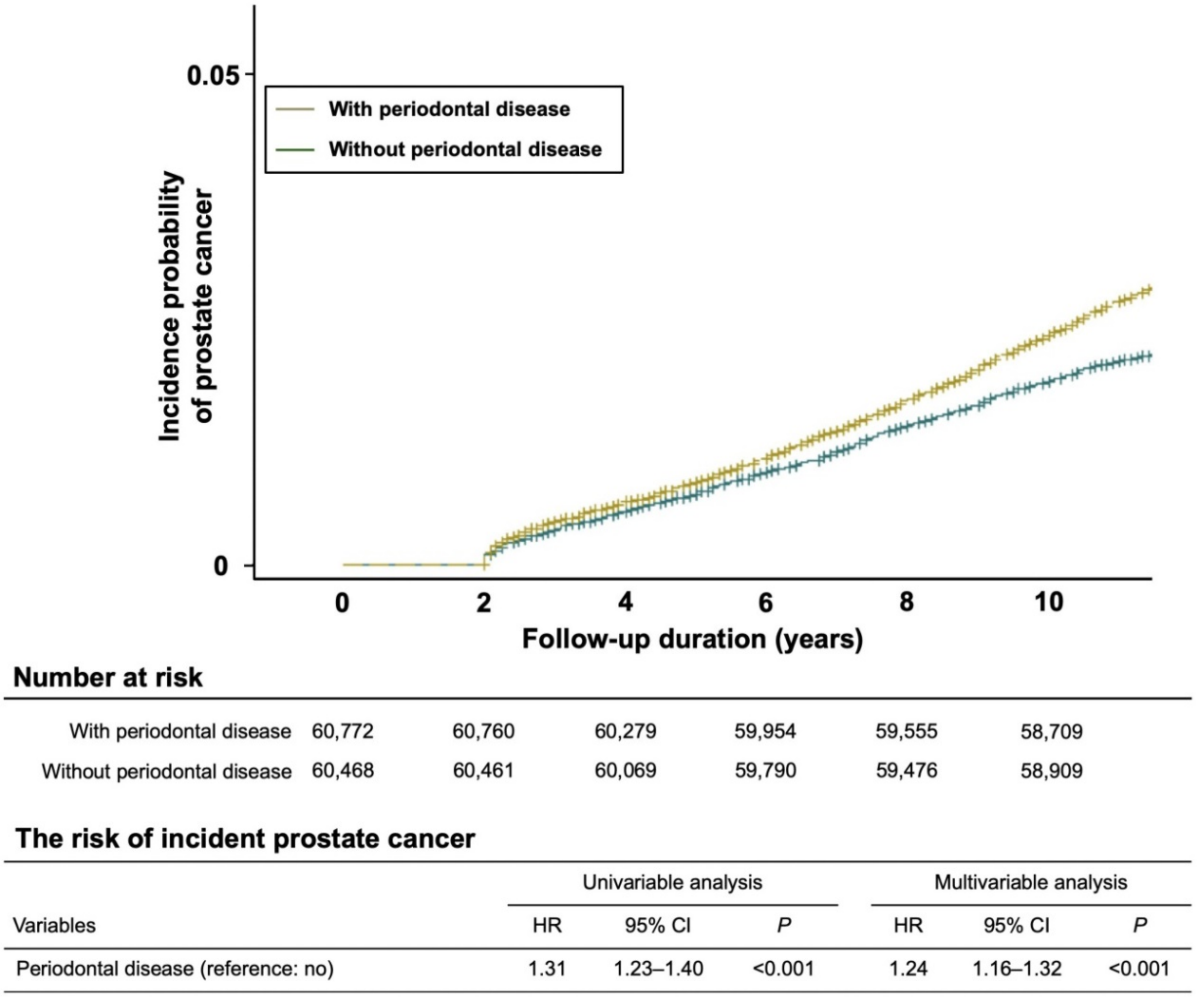
Kaplan-Meier analysis showing the cumulative incidence rates of prostate cancer with and without chronic periodontal disease during the follow-up periods. The risk of incident prostate cancer with chronic periodontal disease was higher and significantly different from that of prostate cancer without chronic periodontal disease (*P* < 0.001).

In the initial study population, 3,622 participants were diagnosed with PC during the follow-up period, and a higher risk of PC incidence was observed in the group with chronic PD than in the group without chronic PD (unadjusted HR 1.31, 95% CI 1.23-1.40, *P* < 0.001; adjusted HR 1.24, 95% CI 1.16-1.32, *P* < 0.001). The Kaplan-Meier curve also showed the risk of incident PC in the groups with and without chronic PD (Figure 2).

## Discussion

In this study, the proportion of PCs among cancers in elderly men was 13.9%, which is similar to that in the World Health Organization GLOBOCAN database that reported 15% of PCs among all male cancers, and these results are also highly consistent with the increase in PC incidence and prevalence at older ages.^6^ Our study, which was conducted on 121,240 men aged ≥ 60 years, showed that the incidence of PC with chronic PD in 10 years was 3.0%, and adjusted HR for developing a new PC was 1.24 (95% CI 1.16-1.32, *P* < 0.001) 15.

Despite the growing interest in oral health and improvement of oral hygiene and dental services, the incidence and prevalence of chronic PD have been consistently increasing as society ages 20. In 2018, PD was the second most common disease in the outpatient hospital care in South Korea, and about 30.1% (15.6 million individuals) of its current population has already received treatment. Previously, chronic PD was defined as a localized and chronic inflammatory response caused by specific anaerobic bacteria in the periodontal pocket, such as *Porphyromonas gingivalis*, *Tannerella forsythia*, *Treponema denticola*, and *Aggregatibacter actinomycetemcomitans*. However, recently, clinical and pathological evidence that chronic PD is closely related to chronic and progressive systemic inflammatory diseases, including cardiovascular disease, diabetes mellitus, rheumatoid arthritis, and osteoporosis, has steadily emerged 21-23.

Recent studies have reported that chronic PD affects the development and/or progression of several types of cancer, including oral and oropharyngeal cancer 16,24. Especially with the exception of orofacial cancer, after adjusting for known risk factors, such as age, sex, and smoking, chronic PD was statistically significantly associated with an increased risk of pancreatic cancer (Michaud et al., HR 1.64, 95% CI 1.19-2.26; Chang et al., HR 1.55, 95% CI 1.02-2.33; Maisonneuve et al., HR 1.74, 95% CI 1.41-2.15) 25-27.

In contrast, few studies have reported positive associations between PC and chronic PD 15,28. In our previous study on 187,934 adult men aged ≥ 40 using data from a South Korean nationwide population based on the NHIS-Health Examinee Cohort, the incidence of PC with chronic PD in 12 years was 0.5%, and adjusted HR for developing a new PC within 12 years was 1.14 (95% CI 1.01-1.31, *P* = 0.042) 15. Another epidemiological cohort study using the Turkish National Cancer Registry database also found that men aged ≥ 35 years with PD were at high risk for developing PC in a follow-up of 12 years (standardized incidence rates 3.75, 95% CI 0.95-10.21, *P* = 0.014) 28.

Dysbiosis of the oral microbiota, bacteria-induced immune evasion and dysregulation, modulation of various signaling pathways, and subsequent inhibition of apoptosis and activation of cell proliferation in patients with chronic PD have all been proposed as pro-tumorigenic mechanisms 29-31. Moreover, some genes (particularly CDKN2B) that are significantly and consistently related with cancer are also associated with PD, which suggests shared genetic susceptibility between the two diseases 28.

Activated inflammatory cells, including neutrophils, macrophages, and dendritic cells, secrete pro-inflammatory and pro-growth substances, such as tumor necrosis factor (TNF)-α, cytokines, chemokines, matrix metalloproteases, and pro-angiogenic molecules. These cells also produce reactive oxygen and nitrogen species, which can induce DNA damage in epithelial cells and produce an environment for both initiation and promotion of carcinogenesis at local and distant sites 32-34. Periodontal pathogens might promote cancer development through invasion of blood vessels, bacteremia, and subclinical infection in distant sites 28.

Among the comorbidities investigated in this study, prostatitis was found to have the closest relationship with PC (crude HR 2.07, 95% CI 1.91-2.25, *P* < 0.001; adjusted HR 1.92, 95% CI 1.77-2.08, *P* < 0.001). Patients with moderate-to-severe prostatitis and chronic PD were found to have higher prostate-specific antigen levels, which are used to track response to androgen deprivation therapy (ADT) for PC 35. Famili et al. found that men with PC undergoing ADT were more likely to have PD than men not undergoing ADT 36. Both PD and prostatitis result in cytokine imbalance toward increased pro-inflammatory cytokines, such as interleukine (IL)-6, IL-8, IL-18, TNF-α, and C-reactive protein, and decreased anti-inflammatory cytokines 37. Additionally, major clinical parameters of PD, especially clinical attachment level, were significantly worse in patients with moderate-to-severe prostatitis 35,37. Considering the similarity in the etiopathogenesis of prostatitis and PD, it is possible that there is a pathological link between them; however, further studies are necessary to draw a conclusion.

The present study has several limitations. First, since all patients with cancer are required to register in the National Cancer Registration and Statistics system, the diagnostic accuracy of PC is highly reliable, while it is difficult to guarantee the diagnostic accuracy of chronic PD due to the nature of this study. Therefore, to increase the diagnostic reliability of chronic PD, diagnosis should be made twice using clinical parameters of the oral health examination, and all participants who did not undergo oral health examination were excluded. Second, because the current NHIS-EC database does not provide information on the stage and grade of PC, it is difficult to confirm the association between the progression or severity of PC and chronic PD. Third, due to the study design, it is hard to distinguish whether PD and PC have a causative relationship or they share the same susceptibility. Finally, since this study has been analyzed retrospectively only for those who have undergone general and oral health examinations, selection bias could occur fundamentally.

## Conclusion

Despite the abovementioned limitations, this is the first study that used NHIS-based elderly cohort data from South Korea, and the results demonstrated a positive and significant association between PC and chronic PD. The retrospective nature of this study limits the conclusiveness of our findings. Therefore, large better controlled studies are warranted to strengthen this evidence of association and help elucidate the underlying biological mechanisms.

## Supplementary Material

Supplementary figures and tables.

## Figures and Tables

**Table 1:** Baseline characteristics of the study population included in the National Health Insurance Service-Elderly Cohort according to the presence and absence of chronic periodontal disease (PD)

Variables	Participants with chronic PD (*n*, %)		Participants without chronic PD (*n*, %)		*P*^a^
Total	60,772	(100.0)	60,468	(100.0)	
**Sociodemographics**					
***Age group (years)***					
60-69	49,822	(82.0)	42,001	(69.5)	< 0.001
≥ 70	10,950	(18.0)	18,467	(30.5)	
***Household income^b^***					
First quintile	9,550	(15.7)	11,063	(18.3)	< 0.001
Second quintile	8,052	(13.2)	9,949	(16.5)	
Third quintile	9,799	(16.1)	10,284	(17.0)	
Fourth quintile	13,785	(22.7)	13,288	(22.0)	
Fifth quintile	19,586	(32.2)	15,884	(26.3)	
***Insurance status***					
MAP	198	(0.3)	351	(0.6)	< 0.001
NHIS (self-employed)	35,658	(58.7)	32,797	(54.2)	
NHIS (employees)	24,916	(41.0)	27,320	(45.2)	
**Comorbidity**					
***CCI***					
0	31,378	(51.6)	32,811	(54.3)	< 0.001
1	15,484	(25.5)	14,454	(23.9)	
≥ 2	13,910	(22.9)	13,203	(21.8)	
***Hypertension***					
Yes	40,660	(66.9)	37,308	(61.7)	< 0.001
No	20,112	(33.1)	23,160	(38.3)	
***Diabetes mellitus***					
Yes	22,761	(37.5)	18,472	(30.5)	< 0.001
No	38,011	(62.5)	41,996	(69.5)	
***Cerebral infarction***					
Yes	12,879	(21.2)	13,216	(21.9)	< 0.001
No	47,893	(78.8)	47,252	(78.1)	
***Angina pectoris***					
Yes	2,553	(4.2)	2,439	(4.0)	0.004
No	58,219	(95.8)	58,029	(96.0)	
***Myocardial infarction***					
Yes	12,321	(20.3)	9,296	(15.4)	< 0.001
No	48,451	(79.7)	51,172	(84.6)	
***Prostatitis***					
Yes	7,144	(11.8)	5,157	(8.5)	< 0.001
No	53,628	(88.2)	55,311	(91.5)	
**Self-reported questionnaire**					
***Smoking status***					
Yes	17,907	(29.5)	19,557	(32.3)	< 0.001
No	42,865	(70.5)	40,911	(67.7)	
***Daily smoking***					
< 20 cigarettes	15,129	(84.5)	16,799	(85.9)	< 0.001
≥ 20 cigarettes	2,778	(15.5)	2,758	(14.1)	
***Alcohol intake habits***					
No drinking	30,304	(49.9)	32,071	(53.0)	< 0.001
1-4 times/week	23,588	(38.8)	20,087	(33.2)	
Almost daily	6,880	(11.3)	8,310	(13.7)	
***One-time alcohol intake***					
< 1 bottle of beer (360 mL)	27,095	(88.9)	25,254	(88.9)	0.992
≥ 1 bottle of beer (360 mL)	3,373	(11.1)	3,143	(11.1)	
***Regular exercise***					
No	32,457	(53.4)	39,529	(65.4)	< 0.001
1-4 times/week	17,623	(29.0)	12,431	(20.6)	
≥ 5 times/week	10,692	(17.6)	8,508	(14.1)	

MAP, Medical Aid Program; NHIS, National Health Insurance Service; CCI, Charlson Comorbidity Index. ^a^*P*-values were obtained using the Pearson chi-square test. ^b^Divided into five quintiles based on the insurance fee imposed on each household, with the MAP group classified into the first quintile.

**Table 2:** Cumulative incidence of prostate cancer in participants with and without chronic PD

Variables	Cumulative incidence of prostate cancer at 10 years				*P*^a^
	Participants with chronic PD (*n*,%)		Participants without chronic PD (*n*,%)		
Total	2,063	(100.0)	1,559	(100.0)	
**Sociodemographics**					
***Age group (years)***					
60-69	1,533	(74.3)	978	(62.7)	< 0.001
≥ 70	530	(25.7)	581	(37.3)	
***Household income***					
First quintile	236	(11.4)	284	(18.2)	< 0.001
Second quintile	227	(11.0)	192	(12.3)	
Third quintile	303	(14.7)	226	(14.5)	
Fourth quintile	489	(23.7)	360	(23.1)	
Fifth quintile	808	(39.2)	497	(31.9)	
***Insurance status***					
MAP	7	(0.3)	13	(0.8)	0.021
NHIS (self-employed)	1,224	(59.3)	871	(55.9)	
NHIS (employees)	832	(40.3)	675	(43.3)	
**Comorbidity**					
***CCI***					
0	907	(44.0)	775	(49.7)	0.001
1	506	(24.5)	364	(23.3)	
≥ 2	650	(31.5)	420	(26.9)	
***Hypertension***					
Yes	1,487	(72.1)	1,033	(66.3)	< 0.001
No	576	(27.9)	526	(33.7)	
***Diabetes mellitus***					
Yes	827	(40.1)	553	(35.5)	0.004
No	1,236	(59.9)	1,006	(64.5)	
***Cerebral infarction***					
Yes	490	(23.8)	331	(21.2)	0.072
No	1,573	(76.2)	1,228	(78.8)	
***Angina pectoris***					
Yes	83	(4.0)	66	(4.2)	0.752
No	1,980	(96.0)	1,493	(95.8)	
***Myocardial infarction***					
Yes	560	(27.1)	303	(19.4)	< 0.001
No	1,503	(72.9)	1,256	(80.6)	
***Prostatitis***					
Yes	465	(22.5)	307	(19.7)	0.038
No	1,598	(77.5)	1,252	(80.3)	
**Self-reported questionnaire**					
***Smoking status***					
Yes	1,061	(51.4)	632	(40.5)	< 0.001
No	1,002	(48.6)	927	(59.5)	
***Daily smoking***					
< 20 cigarettes	871	(82.1)	554	(87.7)	0.023
≥ 20 cigarettes	190	(17.9)	78	(12.3)	
***Alcohol intake habits***					
No drinking	1,137	(55.1)	914	(58.6)	< 0.001
1-4 times/week	753	(36.5)	478	(30.7)	
Almost daily	173	(8.4)	167	(10.7)	
***One-time alcohol intake***					
< 1 bottle of beer (360 mL)	822	(88.8)	580	(89.9)	0.468
≥ 1 bottle of beer (360 mL)	104	(11.2)	65	(10.1)	
***Regular exercise***					
No	1,587	(76.9)	1,154	(74.0)	< 0.001
1-4 times/week	377	(18.3)	181	(11.6)	
≥ 5 times/week	99	(4.8)	224	(14.4)	

^a^*P*-values were obtained using the Pearson chi-square test.

**Table 3:** Univariable and multivariable Cox proportional hazards regression analyses for factors potentially affecting the development of prostate cancer

	Univariable analysis			Multivariable analysis		
Variables	HR	95% CI	*P*	HR	95% CI	*P*
**Sociodemographics**						
***Age group (reference: 60-69 years)***						
≥ 70	1.32	1.23-1.41	<0.001	1.37	1.27-1.48	< 0.001
***Household income (reference: first quintile)***						
Second quintile	0.95	0.83-1.08	0.402	0.99	0.87-1.13	0.855
Third quintile	1.05	0.93-1.18	0.468	1.06	0.94-1.20	0.362
Fourth quintile	1.23	1.10-1.37	<0.001	1.21	1.08-1.35	< 0.001
Fifth quintile	1.40	1.27-1.55	<0.001	1.28	1.15-1.42	< 0.001
***Insurance status (reference: MAP)***						
NHIS (self-employed)	0.77	0.49-1.19	0.237	0.62	0.39-0.96	0.034
NHIS (employees)	0.73	0.47-1.14	0.168	0.62	0.39-0.97	0.036
**Comorbidity**						
CCI (reference: score 0)						
1	1.09	1.00-1.18	0.051	1.03	0.95-1.12	0.441
≥ 2	1.30	1.20-1.40	<0.001	1.19	1.10-1.29	< 0.001
Hypertension (reference: no)	1.23	1.14-1.32	<0.001	1.13	1.05-1.22	0.001
Diabetes mellitus (reference: no)	1.15	1.08-1.23	<0.001	1.08	1.00-1.16	0.041
Cerebral infarction (reference: no)	1.03	0.95-1.11	0.444	0.94	0.87-1.02	0.114
Angina pectoris (reference: no)	1.35	1.25-1.46	<0.001	1.24	1.14-1.34	< 0.001
Myocardial infarction (reference: no)	0.98	0.83-1.15	0.787	0.80	0.68-0.95	0.010
Prostatitis (reference: no)	2.07	1.91-2.25	<0.001	1.92	1.77-2.08	< 0.001
**Self-reported questionnaire**						
Smoking status (reference: no)	1.32	1.22-1.42	<0.001	1.24	1.14-1.33	< 0.001
Daily smoking (reference: <20 cigarettes)	1.18	1.08-1.29	<0.001	1.08	0.98-1.18	0.109
***Alcohol intake habits (reference: no drinking)***						
1-4 times/week	0.88	0.82-0.95	<0.001	0.92	0.86-1.00	0.036
Almost daily	0.70	0.63-0.79	<0.001	0.84	0.74-0.95	0.004
One-time alcohol intake (reference: <1 bottle)	0.87	0.75-1.02	0.076	1.05	0.90-1.24	0.522
***Regular exercise (reference: no)***						
1-4 times/week	0.76	0.70-0.81	<0.001	0.86	0.79-0.93	< 0.001
≥ 5 times/week	0.84	0.71-1.00	0.053	1.08	0.90-1.30	0.428

HR, hazard ratio; CI, confidence interval. Multivariate regression analysis adjusted for age, household income, insurance status, CCI, hypertension, diabetes mellitus, cerebral infarction, angina pectoris, myocardial infarction, prostatitis, smoking status, daily smoking, alcohol intake habits, one-time alcohol intake, and regular exercise.
