# Supplementary material for: Chronic Periodontal Disease increases risk for Prostate Cancer in Elderly individuals in South Korea: a Retrospective Nationwide Population-based Cohort Study
Source: J Cancer. 2020 May 25;11(16):4716–23. doi: 10.7150/jca.45369 (PMC7330688; doi:10.7150/jca.45369)
Supplement: Supplementary file 1 — Supplementary figures and tables. [file jcav11p4716s1.pdf]

Supplementary Material

Supplementary 1. A brief description of the terms related to Charlson comorbidity index

It was mainly extracted from the paper "Charlson industry index as a predictor of periodontal disease in elderly participants" in the Journal of Periodontal and Implant Science), https://doi.org/10.5051/jpis.2018.48.2.92

The Charlson comorbidity index is the most widely used tool for estimating the prognosis of patients with comorbid diseases. The use of this index with weights that vary (from 1 to 6) according to the severity of different diseases was shown to be effective in predicting the 1-year mortality rate based on the following 19 conditions (Table 1):

- 1 point: acute myocardial infarction, congestive heart failure, peripheral vascular disease, cerebral vascular accident, dementia, pulmonary disease, connective tissue disorder, peptic ulcer, liver disease, and diabetes without end-organ damage
- 2 points: diabetes complications, paraplegia, renal disease, and cancer
- 3 points: metastatic cancer and severe liver disease
- 6 points: human immunodeficiency virus.

Table 1. International Classification of Disease, 10th revision (ICD-10) codes and weights for the Charlson comorbidity index scoring system.

Condition	Weighting	Codes (ICD-10)
Acute myocardial infarction[a)]	1	I21, I22, I252
Congestive heart failure	1	I50
Peripheral vascular disease	1	I71, I739, I790, R02, Z958, Z959
Cerebral vascular accident	1	I60-69, G450-452, G454, G458, G459, G46
Dementia	1	F00-F02, F051
Pulmonary disease	1	J40-J47, J61-J67
Connective tissue disorders	1	M32-M35, M058-M060, M063, M069
Peptic ulcer	1	K25-K28
Liver disease	1	K702, K703, K717, K73, K740, K742-K746

Diabetes	1	E101, E105, E109, E111, E115, E119, E131, E135, E139, E141, E145, E149
Diabetes complications	2	E102-104, E112-114, E132-134, E142-144
Paraplegia	2	G041, G81-G822
Renal disease	2	N01, N03, N052-N056, N072, N18, N19, N25
Cancer	2	C0-C96
Metastatic cancer	3	C77-C80
Severe liver disease	3	K721, K729, K766, K767
human immunodeficiency virus	6	B20-B24

[a)]Including coronary artery bypass graft, percutaneous transluminal coronary angioplasty, and angina pectoris.
